# Molecular Responses of Red Ripe Tomato Fruit to Copper Deficiency Stress

**DOI:** 10.3390/plants12102062

**Published:** 2023-05-22

**Authors:** Paco Romero, María Teresa Lafuente

**Affiliations:** Department of Food Biotechnology, Institute of Agrochemistry and Food Technology (IATA-CSIC), Avenida Catedrático Agustín Escardino 7, 46980 Paterna, Valencia, Spain; mtlafuente@iata.csic.es

**Keywords:** COPT, heavy metal, micronutritional stress, phosphate starvation, ripening, *Solanum lycopersicum*, transcriptomics

## Abstract

Fruit nutritional value, plant growth, and yield can be compromised by deficient copper (Cu) bioavailability, which often appears in arable lands. This condition causes low Cu content and modifications in the ripening-associated processes in tomato fruit. This research studies the transcriptomic changes that occur in red ripe tomato fruit grown under suboptimal Cu conditions to shed light on the molecular mechanisms underlying this stress. Comparative RNA-sequencing and functional analyses revealed that Cu deficiency during cultivation activates signals for metal ion transport, cellular redox homeostasis, pyridoxal phosphate binding, and amino acid metabolism while repressing the response to phosphate starvation in harvested fruit. Transcriptomic analyses highlighted a number of novel Cu stress-responsive genes of unknown function and indicated that Cu homeostasis regulation in tomato fruit may involve additional components than those described in model plants. It also studied the regulation of high-affinity Cu transporters and a number of well-known Cu stress-responsive genes during tomato fruit ripening depending on Cu availability, which allowed potential candidates to be targeted for biotechnological improvements in reproductive tissues. We provide the first study characterizing the molecular responses of fruit to Cu deficiency stress for any fruit crop.

## 1. Introduction

Given their sessile nature, plants have developed a wide variety of responses to environmental cues, including those that focus on mitigating both biotic and abiotic stresses. Among abiotic factors, environmental nutrient availability is especially relevant because plants are at the basis of trophic chains and their nutritional deficiencies are often transferred to consumers [1]. In this context, plant resilience depends on metabolic, physiological, and developmental responses to adapt to variations in mineral nutrient availability. The most common plant responses to cope with nutrient scarcity involve the induction and reallocation of specific high-affinity transporters and the accumulation and/or secretion of enzymes and compounds that facilitate nutrient remobilization from soil [2]. Much less is known, however, about the changes that occur in fruits in response to exposure to deficient mineral nutrient stress during growth.

A high percentage of European arable lands are defined as deficient in bioavailable copper (Cu) because of soil alkalization and/or high organic matter contents in their composition [3]. Although plants act as effective miners when Cu is scarce, with severe restrictions they can show diverse deficiency symptoms, which mostly affect fruit production and cause important economic losses in agriculture [1,4,5,6,7]. This is aggravated by the fact that one climate change effect is growing atmospheric CO_2_ levels, which result in greater carbohydrate synthesis in plants and a general loss of their nutritional quality. Indeed, some meta-analyses indicate that Cu and other metal deficiencies would be exacerbated in forthcoming years due to a rise in CO_2_, which would increase problems of obesity and the ‘hidden hunger’ in the world [8,9]. Overall, the integration between Cu homeostasis and the nutritional value of food is needed and will become increasingly relevant in the near future, according to predicted climate change effects, because environmental stress conditions could strongly impact food production and quality. To the best of our knowledge, only Lafuente et al. [10] have recently described how exposing plants to Cu deficiency during cultivation influences the nutritional status and antioxidant capacity of harvested fruit when focusing on tomatoes. Still, there is no information about the components of the Cu homeostasis network in tomatoes or the molecular mechanisms driven by Cu deficiency stress in this fruit.

Plant cells require Cu to maintain essential processes such as photosynthesis, respiration, scavenging ROS, cell wall lignification, response to pathogens, hormone perception, and metabolism [11,12,13]. The dual nature of Cu as an essential micronutrient, but a toxic one in excess, makes a complex homeostasis network necessary whereby Cu content is tightly, but dynamically, regulated [14,15,16]. Cu is soluble in the cupric (Cu^2+^) form but is relatively inert in the cuprous (Cu^+^) state. When available in growth media, Cu^2+^ enters plant root cells through promiscuous divalent cation transporters (YSL, ZIP) [11,17,18]. However, when scarce, plants use a Cu^+^-specific mobilization system based on the acidification of the external medium by H^+^-ATPases, plasma membrane NADPH-dependent cupric reductases (FRO4 and FRO5) [13,19], and the participation of high-affinity Cu^+^ transporters (COPTs) that incorporate Cu^+^ inside cells from the apoplast [12,20,21,22]. In line with this, there is a prevailing dogma of protein-protein interactions that mediate Cu^+^ delivery by which COPT members facilitate Cu^+^ entry across the membrane and modulate its transfer to specific metallochaperones for targeted distribution to different locations and organelles [23,24]. Cu delivery to cytosolic metalloproteins, such as Cu/Zinc (Zn) superoxide dismutases (Cu/ZnSOD) CSD1 and CSD2, is mediated by the Cu chaperone for superoxide dismutase (CCS) [25]. In chloroplasts, the major consumers of Cu in plants, CCS cuprochaperone also delivers Cu to chloroplastic Cu/ZnSOD, Cu^+^ P-type ATPase PAA2, and plastocyanin, among other proteins [26,27,28]. Likewise, CCH and ATOX1 are cuprochaperones that deliver Cu^+^ to the Cu^+^-ATPases located at the endoplasmic reticulum and are more related to ethylene signaling, while COX17 mediates Cu delivery to the cytochrome *c* oxidase (COX) of the respiratory chain in mitochondria [29,30,31,32,33]. By doing so, cuprochaperones minimize the immediate toxicity caused by highly reactive Cu^+^ traffic, which may affect the integrity of membranes and Ca^2+^/K^+^ channels and trigger Ca^2+^- and reactive oxygen species (ROS)-mediated downstream signals [26,34,35].

Therefore, to conciliate dynamic changes in Cu supply and demand and to adapt to environmental metal restrictions or excesses, plants have evolved sophisticated mechanisms that involve a complex homeostatic network to control Cu uptake, delivery to target proteins, and detoxification. In response to Cu scarcity, in a nutshell, plants activate Cu uptake and mobilization and minimize Cu utilization in dispensable pathways by prioritizing Cu for essential processes. In this context, the SQUAMOSA PROMOTER-BINDING-LIKE 7 (SPL7) transcription factor is a key player in promoting Cu uptake under Cu limitation by inducing not only the COPT members located at the plasma membrane [36], but also the expression of iron (Fe) superoxide dismutase FSD1, the CCS chaperone, and several genes that encode laccases and plastocyanin. In addition, SPL7 modulates posttranscriptional regulation events by inducing microRNAs, such as miR398. This, in turn, mediates the mRNA down-regulation of CSD1 and CSD2 [27,36,37]. The substitution of CSD1 and CSD2 for their Fe counterparts, FSD1, when Cu is shortened is considered a hallmark strategy for replacing dispensable Cu proteins with other metalloproteins by performing a similar task. It is surprising that, in spite of its relevance for triggering the Cu-deficiency stress signal and orchestrating the subsequent response, the expression of the SPL7 transcription factor is not induced by Cu limitation. Instead, the SPL7 function is regulated by Cu content throughout conformational changes in its structure, which activate/inactivate its ability to induce the Cu deficiency response [38].

Most of our current understanding of Cu homeostasis regulation comes from research performed on *Arabidopsis* model plants. In horticultural crops, however, research into this topic started a few years ago by identifying and characterizing COPT family members in different species [39,40,41,42,43,44,45,46]. Nevertheless, studies on their regulation under stress conditions and information about other Cu homeostasis network components are still lacking.

Encouraged by our previous data, which demonstrated that exposing tomato plants to low Cu availability during cultivation leads to deficient Cu levels in the peel of harvested fruit [10] and might potentially be transferred to consumers, in the present work we aimed to uncover the molecular mechanisms underlying the Cu deficiency response in tomato fruit by a transcriptomic approach and to focus on a commercial red ripe (RR) mature stage. We found that well-known Cu homeostasis network components, together with a number of proteins of unknown function, were regulated in RR fruit in response to this stress. The results also revealed that adaptation of fruit to low Cu availability might involve the regulation of other nutrients, such as phosphate. In addition, the study of the transcriptional changes of the relevant genes involved in the Cu-stress response, i.e., all the COPT family members, two promiscuous divalent transporters (ZIP and YSL), two cuprochaperones (CCH and ATOX1), and two superoxide dismutases (CSD and FeSOD), in three different ripening stages indicated that Cu homeostasis is modulated during the tomato fruit maturation process, and a Cu-deficient environment during cultivation provokes changes in the regulation of this gene network from the most immature stages.

## 2. Results

### 2.1. Fruit Transcriptomic Changes in Response to Cu-Deficient Growing Conditions

**Figure 1:**
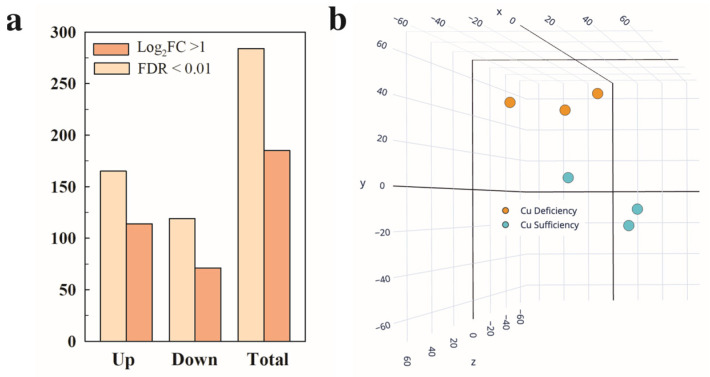
Differential expression and multivariate analysis of tomato fruit in response to Cu deficiency growing conditions. (**a**) Number of differentially expressed genes (DEGs, *P*adj < 0.05) up- and down-regulated in the red ripe fruit harvested from the Cu deficiency conditions in relation to Cu sufficiency. Those DEGs that met an additional cutoff of 2 in the fold change (FC) expression levels (Log_2_ FC ≥ 1) are also indicated. (**b**) Principal component analysis (PCA) based on the total number of DEGs. The three axes in the PCA account for 80.1% of the total variance between the Cu sufficiency and Cu deficiency conditions (X = 36.15%, Y = 28.33%, Z = 15.62%). Three biological replicates from each condition were used for all the analyses.

Given the lower Cu levels found in the pericarp of fruit harvested from plants cultivated under Cu deficiency compared to Cu sufficiency (control) conditions [10], the transcriptome of this tissue was studied in the RR fruit harvested from both environments (Figure 1). In all, 284 differentially expressed genes (DEGs, *P*adj < 0.05) were identified, of which 119 and 165 were up- and down-regulated, respectively, in response to Cu deficiency.

When a cutoff of log_2_FC > 1 (2 in the fold change (FC)) induction/repression levels was set, those numbers were lowered to 71 and 114, respectively (Figure 1a). All the DEGs were used for the principal component analysis (PCA). Two different groups of samples were found that corresponded to the Cu sufficiency and Cu deficiency conditions. The three axes in the PCA accounted for 80.1% of the total variance among conditions (X = 36.15%, Y = 28.33%, and Z = 15.62%). The three biological replicates among each condition were also closely grouped, showing good reproducibility of the transcriptomic results (Figure 1b). It should be noted that in both the Cu sufficiency and Cu deficiency groups of samples, one of the three replicates showed more variability, but despite this, the samples grouped correctly in the PCA, and the statistical analysis (*P*adj < 0.05) provided a reasonable number of DEGs (Figure 1), which supports the robustness of the data.

The full list of DEGs (284 genes) between the RR fruit harvested from the Cu sufficiency and Cu deficiency conditions is shown in Table S1. Of them, the list of the top 10 induced/repressed genes by Cu deficiency is provided in Table 1.

The most induced genes were related to mono- and divalent cation transport and translocation. Thus, the highest induction (~6-fold) was for the high-affinity copper transporter *SlCOPT2*, followed by the cation/H+ exchanger *SlCHX18* (~5-fold) and a gene encoding a calcium-translocating P-type ATPase (~4.8-fold). Then a set of genes with diverse functions, including a WRKY transcription factor, a glycosyl hydrolase, a cytochrome P450, and a chitinase, were induced more than 4.5-fold in this fruit compared to those harvested from the control sufficiency growing conditions (Table 1). It should be noted that the genes encoding for cuprochaperones CCH (*Solyc11g007200*) and ATOX1 (*Solyc05g055310*) were also induced by the Cu stress condition, albeit at lower FC levels (1.9 and 1.8, respectively, Table S1). It is noticeable that two genes of unknown function (*Solyc03g111725* and *Solyc09g097770*) with no orthologous gene within the *Arabidopsis* genome were more than 4-fold induced (Table 1), and 26 more non-annotated genes were induced by Cu deficiency (Table S1). This means that almost 17% (28 out of 165) of the genes induced by this stress were unknown proteins in the tomato genome. Interestingly, the most repressed (~7-fold) gene (*Solyc10g049533*) and three additional genes showing fold change-repression values of about 3.8 (*Solyc01g109100*, *Solyc01g109090,* and *Solyc07g053055*) were also described as unknown proteins (Table 1), and 30 more non annotated genes were repressed by low Cu availability (Table S1). Therefore, more than 28% (34 out of 119) of the repressed genes had an unknown function. On the list of the top 10 repressed genes (Table 1), an inflorescence meristem receptor-like kinase (*IMK2*), a Ser/Thr protein kinase, and diverse genes encoding peptidases, proteases, and hydrolases also appeared. Last, a gene encoding a SPX domain protein (*SPX3*), related to the regulation of phosphate transport and sensing, was more than 3-fold repressed by Cu deficiency.

**Figure 2:**
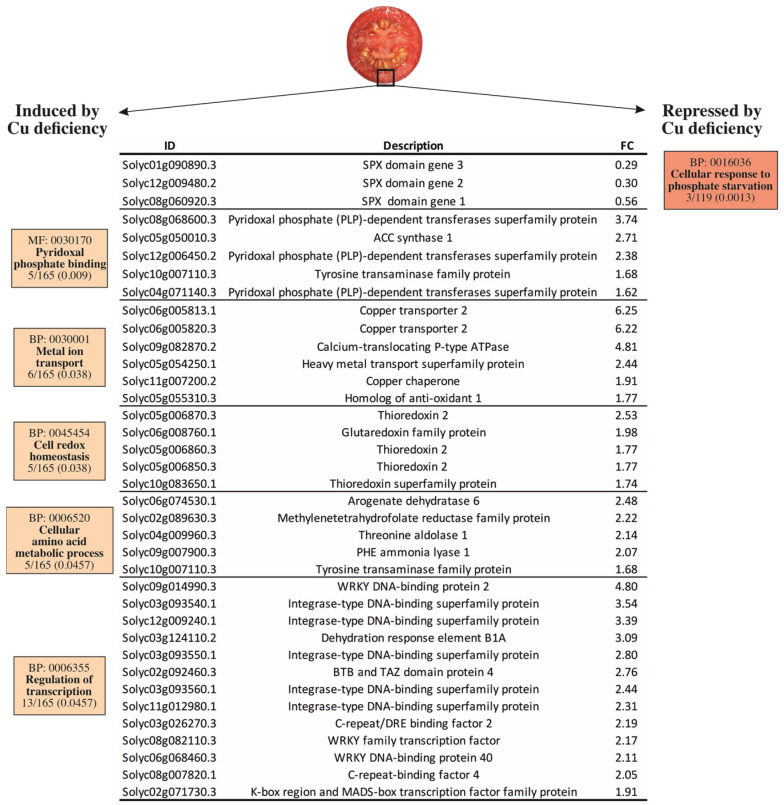
Gene ontology analysis of the differentially expressed genes (DEGs) in the pericarp of the tomato fruit harvested from the plants grown under Cu sufficiency or Cu deficiency conditions. In boxes, the GO code is provided for each molecular function (MF) or biological process (BP), together with the term description, the number of DEGs belonging to that term on the total list of the induced (165) or repressed (119) DEGs, and the adjusted *p*-value (*P*adj < 0.05). ID: Gene identification number according to the iTAG3.2 database; description refers to the annotation of the best *Arabidopsis thaliana* hit for the selected gene. FC: Fold Change.

To narrow down the molecular responses driven by Cu deficiency stress under the growing conditions on the RR tomato fruit, a gene ontology analysis was performed on the total list of DEGs obtained from the transcriptomic comparison. Many biological processes (BPs) were induced by Cu deficiency (Figure S1), of which the most specific are shown in Figure 2. In contrast, the only overrepresented BP in the set of down-regulated genes was ‘cellular response to phosphate starvation’, which contained three *SPX* genes ranging from 1.7- to 3.4-fold change repressions (Figure 2). The ‘pyridoxal phosphate binding’ molecular function (MF) was overrepresented in the set of induced genes. The DEGs included in this MF mostly encode pyridoxal-5′-phosphate (PLP)-dependent transferases and other enzymes that need PLP to function, such as ACC synthase and a tyrosine transaminase protein (Figure 2). Of the induced BPs, ‘metal ion transport’ was especially relevant in the context of this work. This BP included the genes encoding for the SlCOPT2 transporter, a calcium-translocating P-type ATPase, a heavy metal-associated (HMA) Cu transporter protein, and two Cu chaperones (SlCCH and SlATOX1). The ‘cell redox homeostasis’ BP was also overrepresented and composed of several thioredoxin/glutaredoxin family protein members (Figure 2).

The ‘cellular amino acid metabolic process’ and the ‘regulation of transcription’ BPs were overrepresented in the up-regulated genes by Cu deficiency. Belonging to the ‘cellular amino acid metabolic process’ BP, plastid-localized *arogenate dehydratase 6,* involved in phenylalanine biosynthesis, was the most induced gene, while the *phenylalanine ammonia lyase* (*SlPAL*), at the entry point of the phenylpropanoids metabolism, was also ~2-fold induced by Cu deficiency compared to the control fruit. In line with this, a threonine aldolase that catalyzes the condensation between glycine and acetaldehyde and a tyrosine transaminase involved in L-phenylalanine degradation were also induced. A gene encoding a methylenetetrahydrofolate reductase (MTHFR) family protein and involved in methionine metabolism was also included in this BP (Figure 2). Last, several DEGs were grouped in the ‘regulation of transcription’ BP. These genes were mostly integrase-type, WRKY, and C-repeat/DRE DNA-binding transcription factors and ranged from 1.9- to 4.8-fold inductions by Cu stress *versus* the control fruit (Figure 2).

### 2.2. Changes Caused by Low Cu Availability in the Regulation of High-Affinity Cu Transporters (SlCOPTs) during Tomato Fruit Ripening

**Figure 3:**
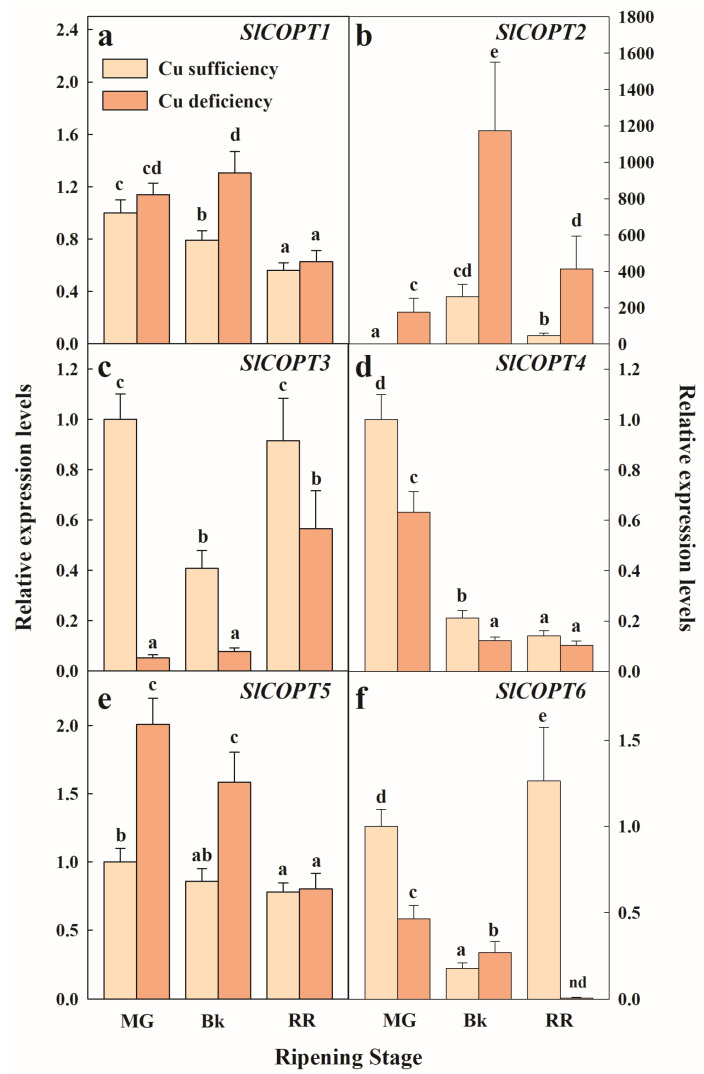
Effect of Cu availability during growth on *SlCOPT*s gene expression in the fruit pericarp tissue at three ripening stages. Bars represent the mean values of three biological replicates ± standard error. Expression levels are relative to those obtained for the MG fruit harvested from the plants grown under Cu sufficiency conditions for *SlCOPT1* (**a**), *SlCOPT2* (**b**), *SlCOPT3* (**c**), *SlCOPT4* (**d**), *SlCOPT5* (**e**) and *SlCOPT6* (**f**), separately. In each panel, different letters indicate significant differences between growing conditions and the fruit ripening stages, according to a two-way ANOVA analysis. *SlCOPT*s are named after their identification within the *Solanum lycopersicum* genome, according to Romero et al. [39]. MG: Mature Green; Bk: Breaker; RR: Red Ripe; nd: not detected expression.

High-affinity Cu transporter family (SlCOPTs) members have been recently identified and characterized in the *Solanum lycopersicum* genome [39]. As *SlCOPT2* was the most induced gene in the pericarp of tomato fruit in response to Cu deficiency growing conditions, we decided to extend this transcriptional study to all the members of this family by also considering different ripening stages (Figure 3).

Under the Cu sufficiency control conditions, *SlCOPT2* expression transiently increased with maturation and peaked in the breaker (Bk) stage (Figure 3b). Conversely, the transcript levels of *SlCOPT3* and *SlCOPT6* showed a transitory decrease in Bk to achieve similar levels to the most immature stage (mature green, MG) in the RR fruit (Figure 3c,f). *SlCOPT1*, *SlCOPT4,* and *SlCOPT5*, however, continuously decreased with fruit maturation under the control conditions (Figure 3a,d,e). Similarly, the genes encoding SlCOPT1, SlCOPT4, SlCOPT5, and SlCOPT6 were repressed with ripening in the fruit grown under Cu deficiency conditions. Contrarily, *SlCOPT3* gene expression increased at the end of the ripening process, and *SlCOPT2* transcripts accumulated with ripening and sharply peaked in the Bk stage (Figure 3).

The expression profile of *SlCOPT2* in the Cu-deficient fruit was parallel to that described under the control conditions but was around 5- to 6-fold higher in any maturity stage. It is also worth noticing in the MG stage that the expression of this gene under Cu deficiency was already ~170-fold higher than under control conditions (Figure 3b). Low Cu availability transiently induced the expression of *SlCOPT1* in Bk (Figure 3a), and that of *SlCOPT5* in the MG and Bk stages (Figure 3e). However, the Cu deficiency condition did not always cause the induction of *SlCOPT*s compared to the control fruit. In fact, the *SlCOPT3* expression levels were consistently lower in the Cu-deficient fruit during ripening (Figure 3c), and the *SlCOPT4* transcript decreased with Cu scarcity in the MG and Bk stages compared to the control fruit (Figure 3d). Similarly, *SlCOPT6* gene expression was mostly repressed by Cu deficiency during ripening compared to the Cu sufficiency conditions, except for a slight induction in Bk (Figure 3f).

### 2.3. Regulation of Cu Stress-Responsive Genes during Tomato Fruit Ripening

**Figure 4:**
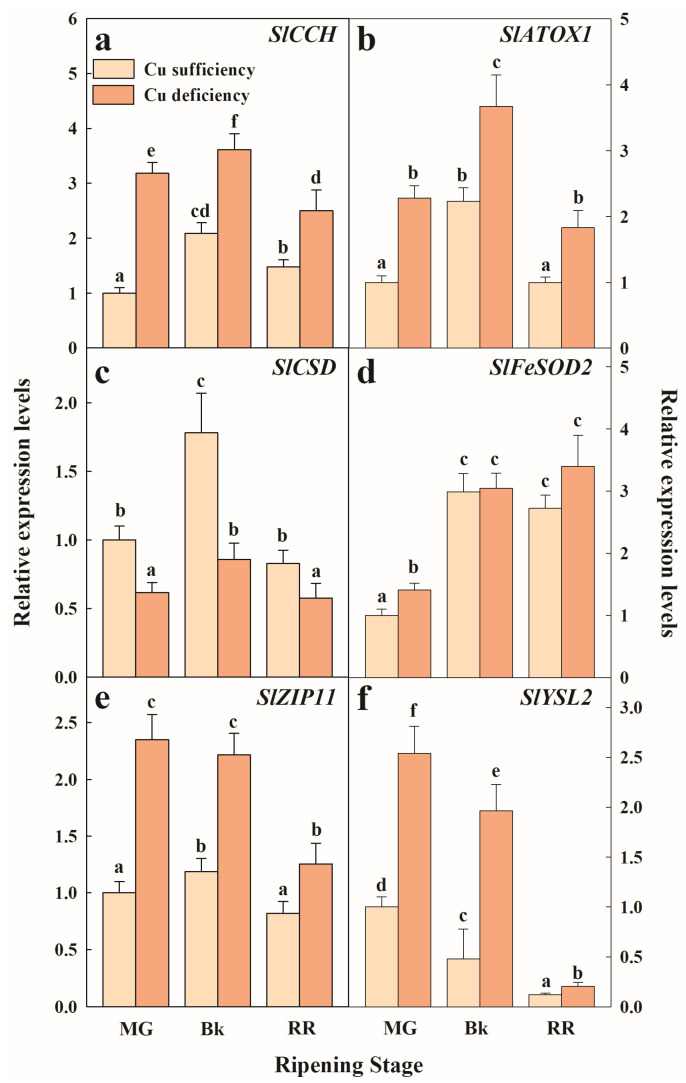
Effect of Cu availability during growth on Cu-homeostasis-related gene expression in the fruit pericarp tissue at three ripening stages. Bars represent the mean values of three biological replicates ± standard error. Expression levels are relative to those obtained for the MG fruit harvested from the plants grown under Cu sufficiency conditions for *SlCCH* (**a**), *SlATOX1* (**b**), *SlCSD* (**c**), *SlFeSOD2* (**d**), *SlZIP11* (**e**) and *SlYSL2* (**f**), separately. In each panel, different letters indicate significant differences between growing conditions and fruit ripening stages, according to a two-way ANOVA analysis. MG: Mature Green; Bk: Breaker; RR: Red Ripe.

In order to better understand the regulation of Cu homeostasis components in tomato, a set of well-known Cu stress-responsive genes was selected, and their expression profiles were studied in tomato fruit in three ripening stages (Figure 4). Because they were included in the ‘metal ion transport’ BP that resulted from the GO analysis (Figure 2), the genes encoding the SlCCH and SlATOX1 cuprochaperones were selected. In addition, two superoxide dismutases (*SlCSD* and *SlFeSOD2*) were included in these analyses because they have been reported to be good molecular markers of Cu stress. Last, two additional unspecific divalent transporters (*SlZIP11* and *SlYSL2*) were also studied.

Our results generally indicated that under either Cu sufficiency control conditions or Cu deficiency stress, the genes related to Cu homeostasis were regulated in tomato fruit during the ripening process. A general expression pattern among the studied genes, except for *SlFeSOD2* and *SlYSL2*, was found in the fruit that developed under Cu sufficiency, which consisted of similar relative expression levels in the most immature (MG) and mature (RR) fruit with a transitory peak in the accumulation of transcripts in the Bk stage (Figure 4). This pattern was also observed when examining the expression of *SICCH, SlATOX1,* and *SlCSD* in the Cu-deficiency-grown fruit (Figure 4). Statistical differences between conditions were still found in all the genes. Hence, the transcript levels of both Cu chaperones *SlCCH* and *SlATOX1* were about 2-fold higher under Cu deficiency in all the ripening stages (Figure 4a,b). Conversely, the gene expression levels of *Cu/Zn superoxide dismutase* (*SlCSD*) were lower during maturation in the fruit exposed to Cu stress compared to the control fruit (Figure 4c). The expression levels of *SlFeSOD2* were, however, the lowest in the MG stage and increased thereafter in the Bk stage, but remained unchanged until the end of the ripening process independently of the Cu condition. The expression levels of the Cu-stressed fruit were higher than in the control fruit, but only in the MG stage (Figure 4d). The transcript accumulation of divalent transporters *SlZIP11* and *SlYSL2* was also influenced by Cu deficiency during ripening. Thus, in spite of *SlZIP11* expression transiently peaking in Bk in the control fruit and remaining steady until the Bk stage to thereafter decrease in the RR fruit under Cu-deficient conditions, transcript levels were always higher in the Cu-stressed fruit (Figure 4e). In contrast, the *SlYSL2* pattern continuously decreased while ripening progressed under both conditions, although expression levels were about 2-fold higher in the Cu-deficient fruit at any maturity stage (Figure 4f).

## 3. Discussion

It is known that low Cu bioavailability leads to reduced growth, distortion, and chlorosis of young leaves and decreases fertility and seed formation in plants and cereal crops [1,17,47,48,49,50,51]. However, much less has been investigated in fruit species. Among the few that have been studied, the effect of Cu limitation during cultivation on the tomato plant phenotype was described decades ago [4,5,6,7], and only recently have the effects of this stress on fruit nutritional value and quality been described [10]. In this work, we identified the genome-wide molecular responses driven by this stress in tomato fruit in the commercial RR stage. To our knowledge, this is the first characterization of the mechanistic basis of Cu deficiency stress in any fruit species by focusing on fruit tissue.

### 3.1. Cu Deficiency Stress Regulates Cu Intake, Mobilisation and Economization during Tomato Fruit Ripening

A key point to controlling Cu homeostasis lies at the level of Cu intake from the apoplast. The comparative transcriptomic analyses between the RR fruit harvested from the plants grown under Cu sufficiency and Cu deficiency conditions revealed that *SlCOPT2*, which is one of the six COPT family members in *Solanum lycopersicum* [39], was the most induced gene in response to Cu stress in the whole tomato genome (Table 1). This gene belongs to the ‘metal ion transport’ BP induced in the RR fruit by Cu-limited conditions (Figure 2). A deeper transcriptional study further demonstrated that all the *SlCOPT* members were regulated throughout the fruit ripening process independently of exposure to Cu stress (Figure 3). Of them, only *SlCOPT1*, *SlCOPT2, SlCOPT5,* and *SlCOPT6* were, at some point, induced by Cu deficiency stress compared to the control conditions. In fact, only the *SlCOPT2* transcript levels were consistently higher under Cu-deficient conditions for the whole maturation process, including the RR stage (Figure 3b). This agrees with findings by Romero et al. [39], who showed that SlCOPT1 and SlCOPT2 were fully able to efficiently transport Cu through the plasma membrane, and both genes were induced by Cu shortage in tomato leaf and root tissues. SlCOPT5 and SlCOPT6 showed much more deficient functionality in spite of their encoding genes also being induced by Cu deficiency in roots and leaves [39]. The present results reveal that, as *SlCOPT5* and *SlCOPT6* are also up-regulated in fruit (Figure 3e,f), their participation in Cu distribution through plant tissues cannot be ruled out. Together, in accordance with studies in model plants [12,14,15,24,52,53,54,55,56], the results presented herein suggest that the induction of Cu uptake by high-affinity Cu transporters is a key response to low Cu bioavailability in tomato, and *SlCOPT2* is a good molecular marker of Cu deficiency stress in this fruit. In this context, it is important to mention that the expression levels of two promiscuous divalent cation transporters, *SlZIP11* and *SlYSL2*, which have been related to Cu and Fe transport and to micronutrients’ stress responses in other species [11,13,17], were also regulated during fruit maturation and induced by Cu deficiency independently of the ripening stage (Figure 4e,f). In addition, the cation/H+ antiporter CHX18, which has been linked with metal and other abiotic stress responses [57,58], was the second most induced gene by Cu scarcity (Table 1). Likewise, a heavy metal transporter (*Solyc05g054250*) and a P-type ATPase (*Solyc09g082870*), both of which belong to the ‘metal ion transport’ BP, were induced by Cu deficiency in the RR fruit (Figure 2). It is important to note that these genes are involved in the acidification and depolarization of the apoplast, which allows Cu^2+^ to reduce into Cu^+^ and be incorporated by COPTs into the cell [11,12,13,53]. These results suggest that Cu shortage during plant growth, which results in low Cu levels in the fruit pericarp [10], would trigger the induction of Cu-specific and general divalent transporters and energy-consuming components of Cu homeostasis to cope with the consequences of this stress condition in fruit.

Besides inducing Cu uptake, exposing tomatoes to limiting Cu availability during plant growth provoked the induction of cuprochaperones in fruit. Indeed, the expression patterns of *SlCCH* and *SlATOX1* under Cu deficiency paralleled those of the control conditions throughout the maturation process, but with expression levels around 2-fold higher (Figure 2 and Figure 4a,b). This is a strategy that probably aims to avoid, to some extent, the oxidative damage caused by Cu^+^ entrance throughout COPT transporters, as reported in other plant species [11,12,13,53]. Further support for this idea comes from the induction of the ‘cell redox homeostasis’ BP in the Cu-deficient fruit (Figure 2), which involves a number of thioredoxin/glutaredoxin family protein members whose functions include ROS scavenging, antioxidation, and Fe-S cluster metabolism [59].

Another important point for controlling Cu homeostasis under metal scarcity is the minimization of Cu utilization in dispensable pathways. Substituting Cu/Zn superoxide dismutase (CSD) for its Fe counterpart (FeSOD) is a hallmark of Cu deficiency stress [12,26,37,60]. Accordingly, the results presented herein indicate that *SlCSD* and *SlFeSOD2* expression patterns vary differently during ripening and in response to Cu stress in tomato fruit. Thus, while *SlCSD* transcript levels remained almost steady and always lower in the Cu-deficient fruit than in the control fruit, *SlFeSOD2* gene expression increased with ripening and was higher under Cu scarcity than under the control conditions in the MG stage (Figure 4c,d), when Fe content is higher in the Cu-deficient fruit [10]. Therefore, these data suggest that tomato fruit economizes Cu availability during ripening through Cu substitution in non-essential metalloproteins by another metal, such as Fe, to perform analogous functions.

### 3.2. Cu Stress Response during Tomato Fruit Ripening Iunvolves the Regulation of Phosphate Homeostasis

Regarding the interaction of Cu homeostasis with other micronutrients [11,56,61,62,63,64], a Cu-Fe interdependence where phosphate (Pi) metabolism might also be involved has been suggested in model plants [55]. Perea-García et al. demonstrated that AtCOPT2 is pivotal for the response to simultaneous Cu and Fe scarcity and plays a part in the antagonistic response of these metals’ deficiency and Pi starvation [55]. Indeed, authors report that the expression of the genes encoding SPX domain proteins, which are molecular markers of Pi-starved plants and participate in Pi homeostasis as phosphate level sensors [65,66,67,68], is induced in *copt2* mutant plants [55]. In agreement with this, our results indicated that the ‘cellular response to Pi starvation’ BP, which included three SPX-coding genes, was repressed in the Cu-deficient fruit (Figure 2). In line with Pi homeostasis, it is also interesting to note the induction of the ‘pyridoxal phosphate binding’ BP by Cu deficiency stress in tomato fruit (Figure 2). Pyridoxal 5’-phosphate (PLP) is considered the active form of vitamin B6, an essential cofactor in amino acid biosynthetic pathways and for a variety of enzymatic reactions like those related to heme/chlorophyll and ethylene synthesis [69]. Of the genes included in this BP, a number of PLP-dependent enzymes were found (Figure 2), such as the ACC synthase involved in ethylene formation. Similarly, several PLP-dependent enzymes belonging to the ‘cellular amino acid metabolic process’ BP were also overrepresented in the set of up-regulated genes by Cu deficiency stress. These enzymes are related to methionine (MTHFR), but mostly to the phenylalanine metabolism. In the last one, it is worth noting the ~2-fold induction by the Cu deficiency of the gene coding for phenylalanine ammonia lyase (*SlPAL*) because PAL was at the entry point of the phenylpropanoids metabolism, which may lead to metabolites performing an important antioxidant function [70]. Altogether, we interpret these results as a strategy of fruit to inhibit the response to Pi starvation under limited Cu availability. As this might be due to increased Pi contents in fruit as a consequence of low Cu accumulation, the aim is to avoid any possible cross-reactions that could trigger cell damage. Moreover, but not excluding them, these responses might be geared to increase the antioxidant capacity of fruit by means of the induction of SPX activity and the accumulation of phenylpropanoids and other secondary metabolites that derive from those amino acids.

Last, the ‘regulation of transcription’ BP, including a large number of transcription factors among which the WRKY and integrase-type DNA-binding proteins predominate, was also induced in the RR fruit in response to Cu deficiency (Figure 2). There is no information about the specific targets of these proteins to allow us to understand their participation in the Cu stress response. However, the marked inductions observed in this set of genes suggest a multitarget transcriptional response by which tomato fruit may attempt to adapt to low Cu levels.

## 4. Materials and Methods

### 4.1. Fruit Material and Growing Conditions

Tomato (*S. lycopersicum* L. cv. Moneymaker) seeds were sterilized and thereafter stratified before growing them in perlite in a greenhouse with 16 h of light at 22 °C and 19 °C at night, as described in [10]. Thirty plants were grouped into two sets of fifteen plants each, and then sorted into three biological replicates of five plants per group. The fifteen plants in the first set were irrigated with a home-made 0.5X Hoagland solution lacking any source of Cu [10] and prepared in demineralized water to produce Cu deficiency. The second set of plants was used as the control (control, +Cu). According to previous reports for this tomato cultivar [39], the watering solution used in the control plants was supplemented with 5 μM CuSO_4_⋅5 H_2_O (Sigma-Aldrich, Madrid, Spain) to generate Cu sufficiency growing conditions. Plants were allowed to grow, and fruit were harvested from each biological replicate in the MG (when fruit had reached their full size, and pulp and peel were entirely green), Bk (when fruit started losing their green color), and RR (when the fruit surface is completely red and displays the expected commercial appearance) ripening stages. The harvested fruit was immediately delivered to the laboratory for further analysis. The pericarp of three fruit from each biological replicate was frozen, homogenized, and stored at −80 °C for further analysis.

### 4.2. RNA Extraction and Gene Expression Analyses

Total RNA was extracted from the pericarp tissue of the fruit harvested in the different above-indicated ripening stages. RNA was extracted with Trizol reagent (Life Technologies, Carlsbad, CA, USA) according to the manufacturer’s directions, as described in [71]. RNA concentration was determined by a NanoDrop ND-1000 spectrophotometer (Thermo Scientific, Wilmington, DE, USA), and its integrity was assessed by agarose gel staining. cDNA was synthesized by following the manufacturer’s instructions in the Maxima H Minus First Strand cDNA Synthesis Kit (ThermoFisher Scientific, Waltham, MA, USA), as in [72]. Real-time quantitative PCRs were carried out as described in [73]. Gene-specific primer pairs (Table S1), 25 ng of cDNA, and SYBR Green qPCR MasterMix (Roche Diagnostics Ltd., Vienna, Austria) were used to generate relative gene expression data in a LightCycler480 System (Roche) instrument. Genes *SlACT, SlCAC, SlSAND,* and *SlRPL2* were used to normalize the expression levels of target genes [74] using the Relative Expression Software Tool software (REST-MC, rest.gene-quantification.info). Values are the means of three biological replicates with two technical replicates ± SD.

### 4.3. RNA-Sequencing, Data Processing and Normalization

Total RNA integrity was assessed in a 2100 Bioanalyzer (Agilent Technologies, Santa Clara, CA, USA) by the RNA 6000 Nano Kit (Agilent). Sequencing libraries were constructed with the TruSeq Stranded mRNA Library Prep Kit^®^ with PolyA selection for ribo depletion (Illumina Inc., San Diego, CA, USA) following the manufacturer’s recommendations and three biological replicates of 2 μg of RNA per RR fruit pericarp sample for both the Cu sufficiency and Cu deficiency conditions. Libraries were sequenced on an Illumina NextSeq 500 platform, and 75-bp single-end reads were generated by the Genome Facility of the SCSIE-UV (Valencia, Spain). Data processing and normalization were performed as in [75]. Briefly, raw sequence reads were checked for quality by the FastP and FastQC software. Clean data were obtained by filtering sequence reads by at least a mean Q28 and mapped to the reference *Solanum lycopersicum* ITAG3.2 genome sequence (Phytozome genome ID: 514) by using the default settings in the TopHat2 v2.1.0 [76] software with the strand-specific alignment type. Only the mapped reads over the protein-coding genes with the RNA-seq quantification pipeline were considered for the raw read counts by assuming opposing strand specificity. The differential expression analysis between the two samples of our experimental design was performed by the edgeR R/Bioconductor package (v3.20.9) [77,78] in the R (v3.4.4) environment [79] based on the negative binomial distribution. Genes were considered DEGs when the significance of the comparison resulted in an adjusted [80] value of *p* ≤ 0.05. The Log_2_ RPM method was followed to estimate the unique gene expression levels. The number of DEGs that met a cutoff of Log_2_ fold change (FC) > 1 was also assessed. A PCA was conducted with the Log_2_ RPM values and was 3D-plotted by plot.ly. The total number of DEGs was used for gene ontology (GO) categorization purposes. The biological process enrichment analysis of DEGs was implemented by the TopGO (v2.30.0) package [81] with the default “weight01” algorithm. A GO term was considered significantly enriched if three DEGs or more were annotated for that term with a classic Fisher value of *p* < 0.05.

### 4.4. Statistical Analyses

The statistical analyses were performed using the STATGRAPHICS software.3. The gene expression data were subjected to analyses of variance (ANOVA), and the significance of the differences was determined by Tukey’s test (*p* < 0.05) on the mean values for all the ripening stages and both growing conditions.

## 5. Conclusions

One important conclusion from this study is that the fruit molecular mechanisms underlying the response to Cu deficiency mostly mimic those described in plants to adapt to this stress condition by focusing on the activation of Cu uptake, the regulation of the redox machinery to avoid oxidative damage, and the minimization of Cu utilization in dispensable pathways by prioritizing Cu utilization for essential processes. Interestingly, the results from the present research work into tomato fruit have revealed for the first time that responses to Cu deficiency may be commonly regulated in fruit, cereal crops, and model plants. This study also unravels a number of genes with unknown function putatively involved in Cu homeostasis, or at least highly responsive to Cu stress, that are uniquely involved in the tomato fruit response. Importantly, this is the case for 17% and 28% of the genes induced and repressed, respectively, in fruit by exposure to Cu scarcity during growth (Table 1 and Table S1). These facts further support the notion that Cu homeostasis in fruits cannot be explained by mere extrapolation from model plants, and new players/responsive genes are yet to be characterized. Further research is still needed to understand the role of these genes in the adaptive mechanisms of fruit to low Cu levels and to assess whether these responses are also found in the tomato plant’s molecular mechanisms driven by this stress.

## Figures and Tables

**Table 1:** List of the top 10 induced and repressed genes in tomato fruit in response to Cu deficiency stress growing conditions.

ID	Description	Fold Change	Log_2_FC	*P*adj	Best *Arabidopsis* Hit
**Induced by Cu deficiency stress**					
Solyc06g005813	Copper transporter 2 (COPT2)	6.25	2.64	0.0009	AT5G59030
Solyc06g005820	Copper transporter 2 (COPT2)	6.22	2.64	0.0010	AT5G59030
Solyc08g081810	Cation/H+ exchanger 18 (CHX18)	4.97	2.31	0.0088	AT5G41610
Solyc09g082870	Calcium-translocating P-type ATPase	4.81	2.27	0.0004	AT3G63380
Solyc09g014990	WRKY DNA-binding protein 2 (WRKY2)	4.80	2.26	0.0000	AT5G56270
Solyc03g111725	Unknown protein	4.78	2.26	0.0017	None
Solyc08g081620	Glycosyl hydrolase 9B14 (GH9B14)	4.77	2.25	0.0037	AT4G09740
Solyc10g080840	Cytochrome P450, family 96, subfamily A, polypeptide 1 (CYP96A1)	4.72	2.24	0.0019	AT2G23180
Solyc03g116225	Basic chitinase (CHI-B)	4.68	2.23	0.0003	AT3G12500
Solyc09g097770	Unknown protein	4.46	2.16	0.0122	None
**Repressed by Cu deficiency stress**					
Solyc10g049533	Unknown protein	0.13	−2.92	0.0000	None
Solyc09g015170	Inflorescence meristem receptor-like kinase 2 (IMK2)	0.19	−2.37	0.0068	AT3G51740
Solyc04g078520	Serine/threonine-protein kinase WNK (With No Lysine)-related	0.25	−2.01	0.0282	AT1G60060
Solyc01g109100	Unknown protein	0.26	−1.97	0.0000	None
Solyc01g109090	Unknown protein	0.27	−1.90	0.0000	None
Solyc04g077630	Serine carboxypeptidase-like 19 (SCPL19)	0.27	−1.89	0.0461	AT5G09640
Solyc07g053055	Unknown protein	0.27	−1.88	0.0047	None
Solyc01g080010	Eukaryotic aspartyl protease family protein	0.28	−1.83	0.0000	AT1G03220
Solyc02g069930	Alpha/beta-Hydrolases superfamily protein	0.28	−1.82	0.0104	AT5G14980
Solyc01g090890	SPX domain gene 3 (SPX3)	0.29	−1.80	0.0006	AT2G45130

## Data Availability

The data presented in this study are available upon request from the corresponding author.

## Supplementary Materials

The following supporting information can be downloaded at: https://www.mdpi.com/article/10.3390/plants12102062/s1, Figure S1: Complete list of the biological processes (BP) overrepresented on the list of up-regulated genes in response to the Cu deficiency conditions in the pericarp of tomato fruit in the RR ripening stage. In the yellow boxes, the GO code is provided for each BP, together with the term description, the number of DEGs belonging to that term on the total list of up-regulated genes, and the adjusted *p*-value (*P*adj < 0.05); Table S1: Total list of the induced (165) and repressed (119) genes in tomato fruit in response to Cu deficiency stress under growing conditions; Table S2: Forward (F) and reverse (R) primers used in this study.
