# Supplementary figures and images for: Molecular Responses of Red Ripe Tomato Fruit to Copper Deficiency Stress
Source: Plants (Basel). 2023 May 22;12(10):2062. doi: 10.3390/plants12102062 (PMC10220619; doi:10.3390/plants12102062)

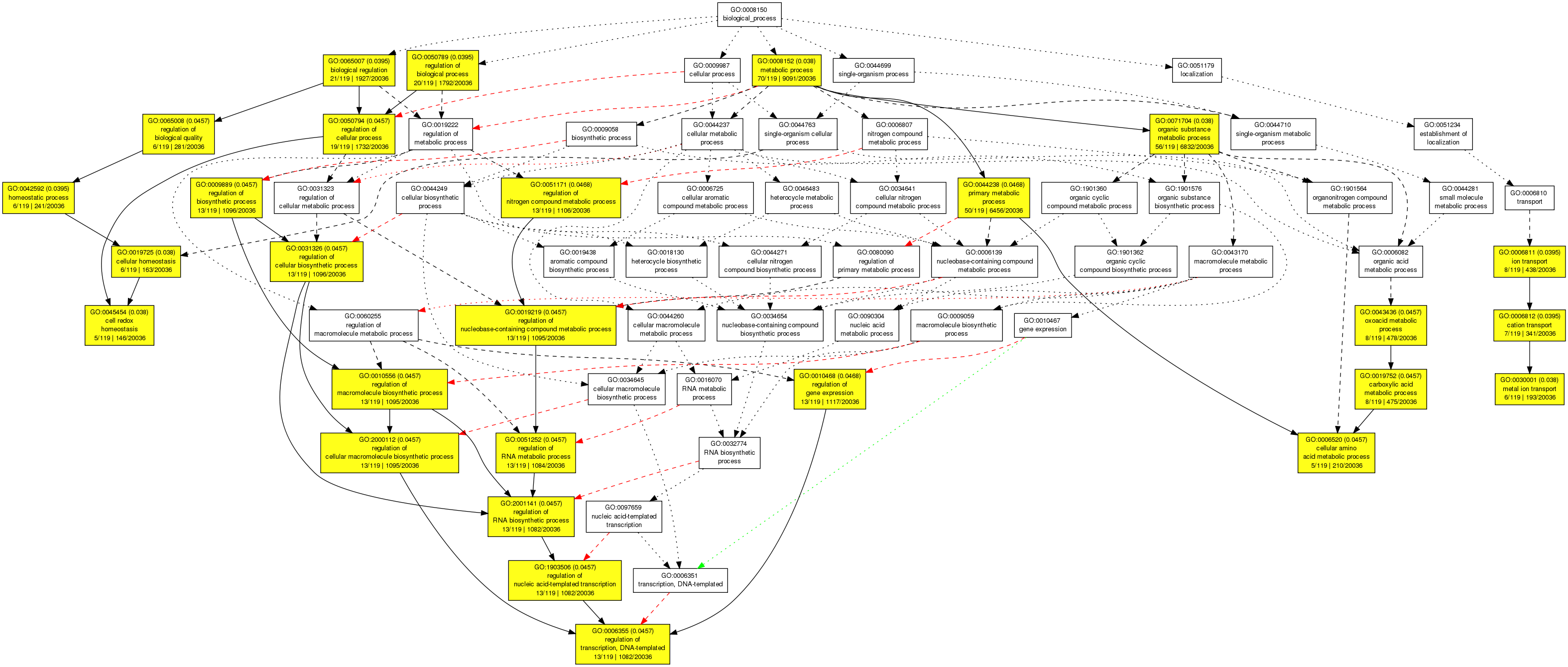

Supplement: Supplementary file 1 [file plants-12-02062-s001.zip › plants-2394309-supplementary/Romero and Lafuente_Supplementary material/Figure S1_Romero and Lafuente_2023.png]
